# The Street Cars and the Public Health

**Published:** 1892-01

**Authors:** 


BUFFALO MEDICAL AND SURGICAL JOURNAL
A MONTHLY REVIEW OF MEDICINE AND SURGERY.

*EDITORS:*
THOMAS LOTHROP, M. D. - - WM. WARREN POTTER, M. D.

All communications, whether of a literary or business character, should be addressed to the managing editor: 284 Franklin Street, Buffalo, N. Y.

Vol. XXXI. JANUARY, 1892. No. 6.

### THE STREET CARS AND THE PUBLIC HEALTH.

We return to this subject, after a somewhat prolonged silence, for the purpose of reminding our readers that the evils heretofore complained of in regard to the street-car service in this city have not been corrected.

Our municipal government is about to inaugurate a new era in the administration of sanitary affairs, and we have very much misjudged the mettle of the new executive, who takes office as Health Commissioner with the beginning of the new year, if we do not find him at once making active endeavors to improve the sanitary condition of the street cars.

The proper management of street cars implies an enforcement of the following conditions: first, absolute cleanliness of each and every car; second, the proper heating of all street cars in Winter; third, no hay or straw to be allowed in any car; fourth, frequent inspection by competent officers, whose duty shall be to see that these regulations are strictly carried out.

We need not enter into an argument as to the propriety of heating the cars from a sanitary standpoint. No person is foolish enough nowadays to claim that heated air is unhealthy air, or that the heating of air necessarily befouls it. Every person with a grain of common sense about such matters knows that a street car can be much better ventilated if it is also heated. Twenty or thirty people crowded into a street car on a frosty morning, without ventilation, and trampling dusty hay or straw, presents a picture of about the worst condition imaginable from a sanitary standpoint. On the other hand, a well-warmed, cleanly car that is properly ventilated with clean rubber matting on the floor, presents a picture of about the nearest approach to perfection that can be obtained from a sanitary standpoint. In these days when so many people are availing themselves of the street cars as a method of transit, it behooves a municipality of the size, intelligence, and importance of Buffalo to exercise the most scrupulous care, and watch with the most jealous eye all avenues that may tend to the propagation or transmission of disease. We have naught but the highest respect for the distinguished gentleman who stands at the head of our street railways in the capacity of president of the corporation, but we certainly have no respect for his unsanitary management of that all-powerful as well as very necessary corporation.

The only wonder is that an intelligent and long-suffering community has endured for so many years the insults that have been daily offered to the special senses of sight, smell, and touch by the uncleanly, unventilated, unheated, and hay-bestrewn street cars that have been tendered as means of transit in Winter to its good-natured inhabitants. But we warn the street car management that the time for deliverance is near at hand ; good nature has been over-taxed, and in our new Health Commissioner we have a man who will not hesitate to take the responsibility of correcting evils that menace the health of the city wherever they may be found.

